# The investigation of thrombocytopenia after transcatheter occlusion of patent ductus arteriosus

**DOI:** 10.1186/s12872-024-03718-0

**Published:** 2024-01-18

**Authors:** Jipei Wang, Xiaoming Wang, Xuefei Xu, Limin Xie, Pengwei Yang

**Affiliations:** Department of Cardiovascular Medicine, Henan Provincial Chest Hospital, Zhengzhou University, No. 1, Weiwu Road, Zhengzhou, Henan 450000 China

**Keywords:** Patent ductus arteriosus, Thrombocytopenia, Transcatheter occlusion, Complication

## Abstract

**Objective:**

To investigate the risk factors for thrombocytopenia after transcatheter occlusion operation of patent ductus arteriosus (PDA).

**Method:**

Retrospective analyses were conducted using clinical data from 106 patients with PDA who underwent transcatheter closure operations at Henan Provincial Chest Hospital, Zhengzhou University, from January 2018 to June 2022. The study compared the changes in platelet counts before and after the operation, and investigated the risk factors for thrombocytopenia following PDA closure in different groups and layers.

**Results:**

The platelet count of patients with PDA significantly decreased after undergoing transcatheter PDA occlusion. Logistic regression analysis revealed that factors such as PDA diameter, occluder diameter, pressure difference on the two sides of the occluder, and residual shunt were associated with an increased risk of thrombocytopenia following PDA occlusion. Specifically, the size of the occluder and the pressure difference between the two sides of the occluder were found to have a negative correlation with the postoperative platelet count. Further subgroup analysis demonstrated that the incidence of total thrombocytopenia was significantly higher in the large PDA group compared to the small-medium PDA groups.

**Conclusion:**

Our findings suggest that occluder diameter, the pressure difference between the two sides of the occluder, and the residual shunt are major risk factors correlated with the incidence of postoperative thrombocytopenia. However, a multicenter and long-term prospective study is required to further evaluate the prognosis of PDA patients with thrombocytopenia after transcatheter occlusion.

## Introduction

Patent ductus arteriosus (PDA) is a common congenital cardiovascular malformation, accounting for 10–21% of congenital heart disease, and it is predominantly observed in women [1]. PDA is a significant risk factor that increases the incidence rate of premature delivery of infants in pregnant female patients, with the incidence rate being as high as 80% for infants weighing less than 1 kg [2, 3]. Since the surgical treatment for PDA was first implemented by Gross et al. in 1938 [4], surgical ligation has been widely recognized as the primary approach for PDA patients. However, surgical operation presents several challenges, including greater trauma, anesthesia accidents, blood transfusion reactions, incision scars, and perioperative complications [5]. With the advancements in interventional technology and occluders, transcatheter occlusion procedures have shown an increasing success rate in PDA intervention [6]. Currently, interventional transcatheter occlusion has replaced surgery as the preferred choice for PDA management due to its advantages, such as the absence of incision scars, lower risk of trauma, positive outcomes, and faster recovery [7].

Although the transcatheter occlusion approach reduces potential trauma in the artery, several postoperative complications were observed in clinics, such as arrhythmia, thrombus, valve regurgitation and occluder dislocation [8, 9]. With the widespread application of occlusion intervention, postoperative thrombocytopenia has become one of the most common complications. The occurence of thrombocytopenia could increase the risk of hemorrhagic and ischemic events, prolong the ICU stay and reduce the survival rate [10–13]. Besides, the incidence of severe thrombocytopenia in PDA patients after transcatheter occlusion operation can reach up to 1.39% [14]. In a study involving 5 PDA patients aged from 12 to 42 years old, all patients experienced postoperative thrombocytopenia after transcatheter occlusion [15]. Postoperative shunts were found in these 5 patients, and the lowest platelet counts were observed 4–7 days after transcatheter closure operation. In another study with a larger cohort size (*n* = 336), about 70.8% of patients developed a mild level of thrombocytopenia (platelet count reduced by 10–49%) and 6.3% of the enrolled subjects displayed severe thrombocytopenia (platelet count reduced by more than 50%), suggesting that thrombocytopenia could be common consequence after transcatheter closure operation [16]. Moreover, patients with severe thrombocytopenia were associated with prolonged hospitalization, suggesting that improving thrombocytopenia could enhance the recovery of PDA patients after transcatheter closure.

Although there have been reports on the incidence of postoperative thrombocytopenia in patients with PDA, there is a lack of comprehensive investigation on the incidence, change pattern of platelet counts, potential risk factors and mechanisms. The clinical prognosis of thrombocytopenia after transcatheter occlusion surgery also warrants further clarification. In this study, we conducted retrospective analyses on 106 patients with PDA who underwent transcatheter closure operations. We analyzed the pattern of platelet count changes before and after the operation and assessed the potential risk factors for postoperative thrombocytopenia using logistic regression. Our data could provide insights into optimizing transcatheter closure operations to reduce the risk of postoperative thrombocytopenia.

## Methods

### Subjects

The study enrolled patients with PDA who were hospitalized in the Cardiovascular Department of Henan Provincial Chest Hospital, Zhengzhou University and underwent transcatheter closure operation between January 2018 and June 2022. The inclusion criteria were based on the Chinese guidelines for interventional treatment of congenital heart disease. Indications for PDA interventional therapy were categorized as follows: Class I—patients with clinical symptoms and weight ≥ 8 kg; Class II—patients with body weight between 4 and 8 kg, with clinical symptoms or continuous cardiac murmur; Class III- patients with PDA diameter ≥ 14 mm, 3 months after control of infective endocarditis; Class IV-PDA with mild to moderate mitral or aortic stenosis and insufficiency. Exclusion criteria were as follows: endocarditis with vegetations in the heart, resistive pulmonary hypertension, intracardiac malformations not suitable for interventional treatment, other contraindications for operation and cardiac catheterization, predisposition to thrombotic events for interventional therapy, and PDA dependent intracardiac malformations. All enrolled patients provided informed consent for interventional closure therapy, and the study was approved by the Ethics Committee of our hospital.

### Data collection and definition

The basic clincial characteristics of the patients were collected, including age, sex, weight, PDA diameter, occluder diameter, pressure on both sides of the occluder, heparin dosage during the operation, residual shunt, preoperative blood routine examination, coagulation function, liver and kidney function, transthoracic echocardiography and electrocardiogram. A blood routine examination was conducted 1–2 days after the operation, and echocardiogram was recorded 24 h after the operation. The study included 28 cases in the large PDA group and 78 cases in the small-medium PDA group. The large PDA group consisted of children weighing less than 8 kg with a narrowest diameter of the PDA ≥ 6 mm, and adults with a PDA diameter ≥ 10 mm. Small-medium group consisted of the children with a PDA diameter < 6 mm and adults with a PDA diameter < 10 mm.

Thrombocytopenia grades were defined as follows: thrombocytopenia (platelet count < 100 × 10^9^/L), mild thrombocytopenia (≥ 50 × 10^9/^L), moderate thrombocytopenia (30 × 10^9^/L − 50 × 10^9^/L), and severe thrombocytopenia (< 30 × 10^9^/L) [17]. Bleeding severity grades were categorized as follows: Grade 0 (no bleeding), Grade 1 (skin ecchymosis and ecchymosis, epistaxis < 1 h, gingival bleeding), Grade 2 (bleeding visible to the naked eye, no need for transfusion of suspended red blood cells), Grade 3 (bleeding requiring blood transfusion correction), and Grade 4 (fatal bleeding). Mild bleeding was defined as Grade 1 and 2, while severe bleeding was defined as Grade 3 and 4 [18].

### Transcatheter occlusion procedure

PDA blocking method: The puncture site was disinfected, and the femoral artery and vein were punctured to create a sheath after anesthesia. The size of the PDA was determined through descending aorta angiography, and the pressures on the descending aorta side and the pulmonary artery side were recorded. Once the catheter was directed to the pulmonary artery, a guide wire was passed through the PDA to enter the descending aorta to establish a delivery track. All the occluders used for the operations were produced by Shanghai Shape Memory Alloy Co., Ltd. (Shanghai, China). The waist diameter of duct occluder used is 3 ~ 6 mm larger than the PDA diameter measured under angiography. The occluder was installed into the descending aorta side through the delivery track. The distal fimbria of the occluder was opened, and the occluder was slowly withdrawn to the aortic end and positioned close to the pulmonary artery side of the PDA. The waist of occluder was released until the posterior end of the occluder reached the pulmonary artery. A pulling test was performed to ensure that the occluder was firmly fixed without shifting. After several minutes, angiography of the descending aorta was performed to observe the suitability of the occluder’s location and determine any residual shunt. If no obvious abnormalities were found, the occluder would be released and the pressures on the descending aorta side and pulmonary side would be measured again. After the occluder deployment, Trans-Thoracic Echocardiography was performed to evaluate occluder position, possible procedural complications, and residual shunts. The puncture sheath was then removed and the puncture site was bandaged. During the operation unfractionated heparin was initially administered at a dosage of 100U/kg, and additional 1000 U heparin was further administered for every 1 h of extension of operational procedure.

### Treatment after transcatheter occlusion procedure

Routine blood examinations were conducted for 2 consecutive days after interventional closure. Venous blood was drawn at 6:00 a.m. every morning after overnight fasting. Cardiac ultrasound was rechecked 24 h after the operation. Patients with a decreased platelet count were continuously monitored. If the platelet count was slightly reduced, intravenous instillation of dexamethasone was administered. In cases of bleeding, patients received platelet transfusions from apheresis. For patients with a severe decrease in platelet count, active platelet transfusion was performed. Daily tests including blood tests, stool routine tests, urine routine tests, and blood coagulation function tests were conducted until the platelet count returned to the normal range. If no thrombocytopenia was found 2 days after the operation, routine blood examination was repeated 1 week later to verify whether there was a thrombocytopenia.

### Statistical analysis

SPSS 18.0 software was utilized for data processing and analysis. The measurement data were presented as mean ± standard deviation (SD) and analyzed for statistical significance using a student’s t-test. The count and rate analysis data were analyzed using a chi-square test. A one-way ANOVA was conducted to compare the three groups. Correlation analysis was performed to examine the relationship between the diameter of the occluder, postoperative platelet count, pressure difference across the PDA occluder, and decrease in platelet count. Logistic regression analysis was used to identify the risk factors of thrombocytopenia after PDA, with age, sex, body weight, PDA diameter, occluder diameter, pressure difference on both sides of the occluder, amount of heparin used during the operation, residual shunt, and eosinophil count as independent variables and thrombocytopenia as the dependent variable. A *p*-value less than 0.05 was considered statistically significant.

## Results

### Demography of enrolled patients

A total number of 106 patients diagnosed with PDA were enrolled in the study, including 27 males and 79 females. The average age was 12.83 ± 12.89 years, weight was 26.60 ± 16.17 kg, height was 123.76 ± 27.03 cm, PDA diameter was 5.80 ± 2.63 mm, and preoperative platelet count was (263.93 ± 89.99) × 10^9^/L (refer to Table 1 for dilated information).

**Table 1:** Baseline characteristics of enrolled patients

Parameter	PDA patients(*n* = 106)
Gender (male/female)	27/79
Age(year)	12.83 ± 12.89; 1 year old to 64 years old
Height(cm)	123.76 ± 27.03
Weight(kg)	26.60 ± 16.17
PDA diameter(mm)	5.80 ± 2.63
Preoperative platelet count(x10^9^/L)	263.93 ± 89.99
Eosinophil count(x10^9^/L)	0.176 ± 0.14
White blood cell count(x10^9^/L)	7.30 ± 2.38
Red blood cell count(x10^12^/L)	4.57 ± 0.47
Hemoglobin(g/L)	128.04 ± 18.77
International normalized ratio	0.93 ± 0.09
ProthrombinTime(PT)(second)	12.42 ± 1.08
Activated partial thromboplastin time(APTT)(second)	32.95 ± 6.72

### Platelet count and degree of bleeding before and after transcatheter occlusion

The changes of platelet count before and after transcatheter occlusion was summarized in Table 2. The platelet count after transcatheter closure was (202.89 ± 104.33) × 10^9^/L, which was significantly lower compared with the preoperative count (263.93 ± 89.99) × 10^9^/L. After transcatheter closure, 6 patients experienced mild thrombocytopenia (5.66%), 8 patients showed moderate thrombocytopenia (7.55%), and 7 patients displayed severe thrombocytopenia (6.60%). Mild bleeding occurred in 9 patients (8.49%) after transcatheter closure, and no severe bleeding was observed in any of the patients.

**Table 2:** Changes of platelet count and degree of bleeding before and after transcatheter closure

	PDA (*n* = 106)	Large PDA (*n* = 28)	Small-medium PDA (*n* = 78)
Preoperative platelet count /(×10^9^ /L)	263.93 ± 89.99	230.29 ± 80.17	276.01 ± 86.64
Postoperative platelet count /(×10^9^ /L)	202.89 ± 104.33	107.78 ± 94.61	237.04 ± 85.05
Platelet count decrease (%)	19.81%(21)	64.29%(18)	3.85%(3)
Milde platelet count decrease (%)	5.66%(6)	14.29%(4)	2.56% (2)
Moderate platelet count decrease (%)	7.55%(8)	25.00%(7)	1.28%(1)
Severe platelet count decrease (%)	6.60%(7)	25.00% (7)	0%(0)
Mild bleeding (%)	8.49%(9)	28.57%(8)	1.28%(1)
Severe bleeding (%)	0%(0)	0%(0)	0%(0)

### Risk factors related to thrombocytopenia in PDA patients after occlusion

#### Comparison of basic characteristics between thrombocytopenia group and normal platelet group after PDA occlusion

The comparison of the basic characteristics of patients in the thrombocytopenia group and the normal platelet count group after PDA occlusion was summarized in Table 3. In the thrombocytopenia group, the diameter of the PDA, the diameter of the occluder, the pressure difference between the two sides of the occluder, and the percentage of residual shunt were significantly higher when compared to the normal platelet group (*P* < 0.05). However, there were no statistically significant differences in age, sex, weight, intraoperative heparin consumption, or eosinophil count between two groups (*P* > 0.05).

**Table 3:** Comparison of basic characteristics between thrombocytopenia group and normal platelet group after PDA occlusion

Item	Thrombocytopenia group(*n* = 21)	Normal platelet count group(*n* = 85)	P value
Age(year)	13.3 ± 7.6	12.1 ± 7.8	0.604
Gender(male/female)	3/18	24/61	0.193
Weight(kg)	28.48 ± 14.17	26.14 ± 16.67	0.556
PDA diameter(mm)	9.07 ± 1.76	5.00 ± 2.13	0.001
Occluder diameter(mm) Pressure difference on	18.57 ± 3.96 116.07 ± 32.07	10.65 ± 3.32 79.27 ± 21.83	0.001 0.001
occluder sides(mmHg)			
Intraoperative heparin dosage(U)	2895.23 ± 1370.94	2614.12 ± 1667. 24	0.476
Residual shunt(no/yes)	14/7	85/0	0.005
Eosinophil count(×10^9^/L)	0.16 ± 0.11	0.18 ± 0.14	0.509

#### Logistic regression analysis on risk factors of thrombocytopenia after PDA occlusion

**Fig. 1:**
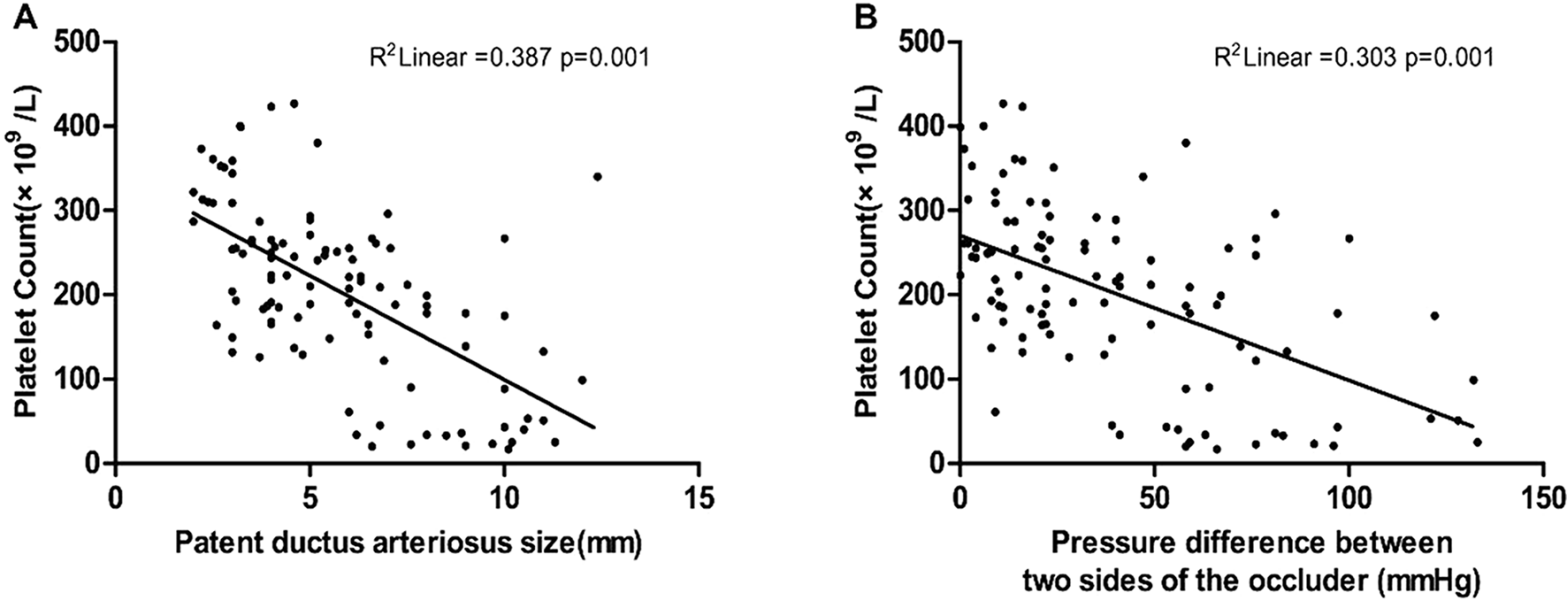
Relationship between platelet count, PDA diameter and pressure difference on both sides of occluder

The logistic regression analyses in Table 4 revealed that several risk factors were significantly associated with thrombocytopenia after PDA closure. These risk factors included the defective diameter of PDA, the diameter of the occluder, the pressure difference between the two sides of the occluder, and the residual shunt (*P* < 0.05). However, there was no significant association between age, sex, weight, heparin dosage, eosinophil count, and thrombocytopenia after PDA closure (*P* > 0.05). The linear correlation analysis in Fig. 1 demonstrated that both the PDA diameter and the differential pressure on both sides of occluder were negatively correlated with thrombocytopenia. The correlation coefficient for the PDA diameter was *R*=-0.622 (R^2^ = 0.387, *p* = 0.001, Fig. 1A), while the correlation coefficient for the differential pressure was *R*=-0.55 (R^2^ = 0.303, *p* = 0.001, Fig. 1B).

**Table 4:** Logistic regression analysis of risk factors of thrombocytopenia after PDA occlusion

Risk factor	Regression coefficient	Standard error	Wald value	OR	95% CI	P value
Age	0.354	0.277	1.632	1.424	0.828 ~ 2.449	0.201
Gender	0.859	0.669	1.651	2.361	0.637 ~ 8.752	0.199
Weight	0.491	0.333	2.182	1.635	0.852 ~ 3.137	0.14
PDA diameter	3.507	0.702	24.966	33.333	8.424 ~ 131.898	0.001
Occluder diameter	4.227	1.085	15.187	68.508	8.169 ~ 574.502	0.001
Pressure difference on occluder sides	0.046	0.014	10.455	1.047	1.018 ~ 1.076	0.001
Heparin dosage	0	0	0.515	1	1.000 ~ 1.000	0.473
Residual shunt	1.173	0.278	20.069	4.929	2.539 ~ 4.604	0.001
Eosinophil count	0.958	0.512	3.496	2.605	0.955 ~ 7.108	0.999

### Comparative analysis of subgroups according to PDA diameter

#### Comparison of postoperative thrombocytopenia between large PDA group and small-medium PDA group

The incidence of mild thrombocytopenia was 14.29% (4 cases) in the large PDA group after transcatheter closure. Moderate thrombocytopenia was observed in 25.00% (7 cases), and severe thrombocytopenia in 25.00% (7 cases) in the same group. In the small-medium PDA group, the incidence of mild thrombocytopenia was 2.56% (2 cases), moderate thrombocytopenia was 1.28% (1 case), and there were no cases of severe thrombocytopenia. The total incidence of thrombocytopenia in the large PDA group was significantly higher than that in the small-medium PDA group (*P* < 0.05), as shown in Table 2.

#### Comparison of PDA diameter, occluder diameter and occluder side pressure in two subgroups

The comparative analysis of PDA diameter, occluder diameter, and the side pressure of the occluder between the large PDA group and small-medium PDA group were summarized in Table 5. All these parameters exhibited significantly higher values in the large PDA group compared to the small-medium PDA group, indicating that interventions for large PDA present greater challenges.

**Table 5:** Comparison of PDA diameter, occluder diameter and side pressure of occluder between large PDA group and small-medium PDA group

Item	Large PDA group	Samll-medium PDAgroup	T value	P value
PDA diameter (mm)	8.93 ± 1.90*	4.58 ± 1.67	13.97	0
Occluder diameter (mm)	16.47 ± 3.65*	10.29 ± 3.30	10.11	0.001
Occluder left pressure (mmHg)	154.20 ± 14.80*	122.35 ± 14.32	12.19	0.008
Occluder left pressure (mmHg)	42.87 ± 9.43*	34.33 ± 8.21	5.68	0.001
Pressure difference on two sides (mmHg)	111.34 ± 11.64*	88.03 ± 10.56	11.93	0.002

*p < 0.05

### Predictive potential of PDA diameter and occluder diameter for postoperative thrombocytopenia

**Fig. 2:**
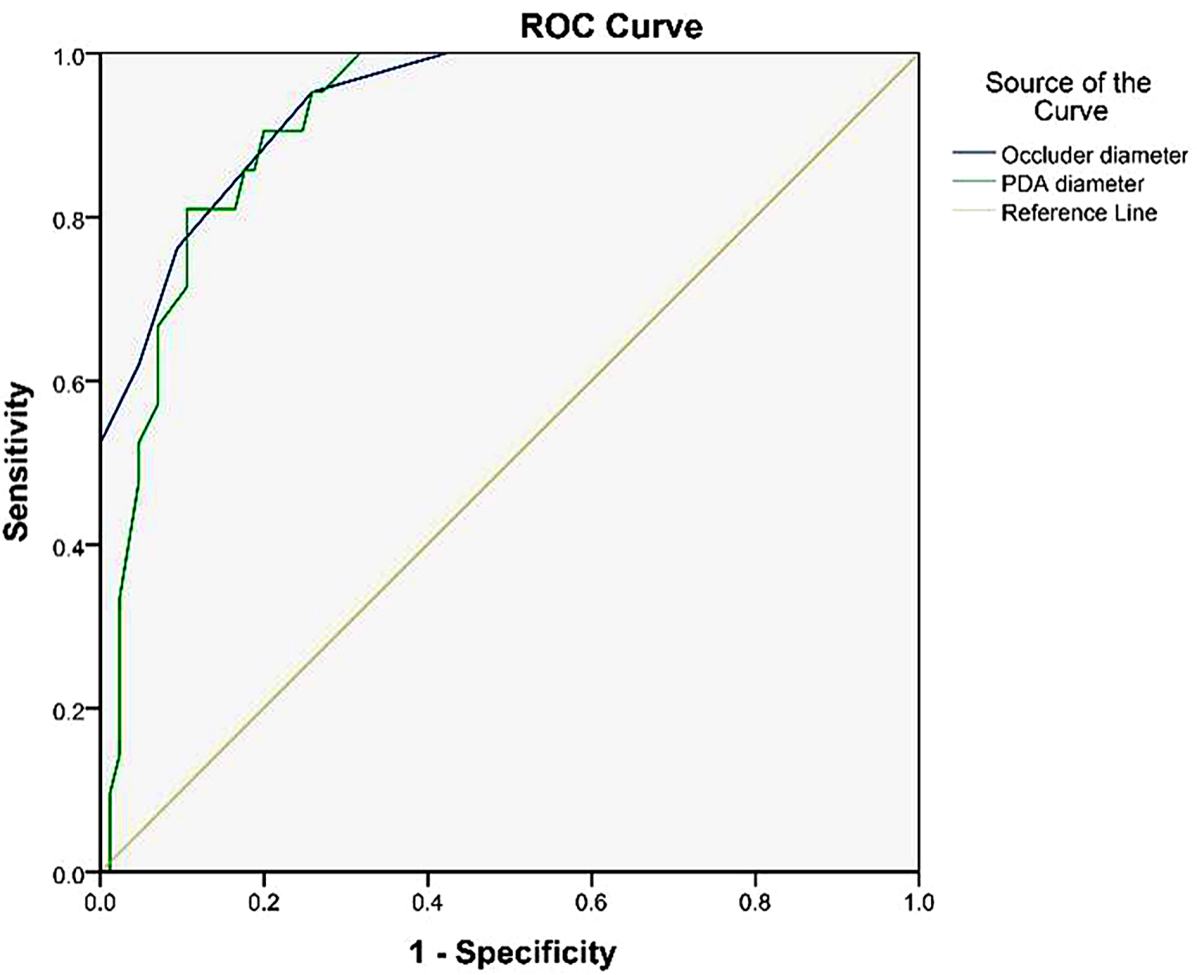
Predictive value of PDA defect diameter and occluder diameter for thrombocytopenia in PDA patients after occlusion surgery

The predictive value of PDA defect diameter and occluder diameter in postoperative thrombocytopenia was evaluated by ROC curve analysis (Fig. 2). The area under the ROC curve for assessing postoperative thrombocytopenia in PDA patients after occlusion was 0.919 (*p* < 0.001) based on the diameter of the PDA defect. The optimal cut-off point for predicting poor prognosis was 6.55 mm, with a sensitivity of 90.5% and a specificity of 80.0%. The area under the ROC curve for determining postoperative thrombocytopenia based on the occluder diameter is 0.938 (*p* < 0.001), and the optimal cut-off point for judging poor prognosis is 13.00 mm. The sensitivity for judging poor prognosis is 95.2%, and the specificity is 74.1%. These data suggest the predictive potential of PDA diameter and occluder diameter for thrombocytopenia in PDA pateints after transcatheter occlusion.

## Discussion

The majority of PDA patients can now be successfully treated with transcatheter occlusion. However, a common postoperative risk factor is a decrease in platelet count, particularly in cases of large PDA occlusion. Our study identified several risk factors for thrombocytopenia after PDA occlusion, including the defective PDA diameter, occluder diameter, pressure difference between the two sides of the occluder, and residual shunt. Severe thrombocytopenia, often accompanied by bleeding events, was more prevalent in patients with large PDAs. Additionally, we observed a negative correlation between platelet count and occluder diameter after PDA occlusion.

Thrombocytopenia after PDA interventional closure has been found to be associated with the diameter of the occluder [16]. There is a positive correlation between thrombocytopenia and the contact area of the occluder and blood vessel. A larger occluder diameter results in more platelets adhering to and gathering in the occluder umbrella, leading to a greater reduction in platelet count [19, 20]. Additionally, we observed a negative correlation between the pressure difference across the occluder and the decrease in platelet count after PDA occlusion. This could be attributed to the high-speed blood flow induced by the high pressure difference, which flushes from the high-pressure aortic surface to the low-pressure pulmonary artery through the small cavity in the polyester filler of the occluder. Platelets possess strong adhesion and aggregation capabilities. When they enter the small cavity of the occluder, they can adhere and aggregate, forming platelet thrombus. However, the high-speed blood flow may cause deformation, damage, or fragmentation of the platelet thrombus, leading to its washout. Subsequently, new platelets adhere to the polyester component of the occluder and initiate a cycle of platelet thrombus formation and fragmentation. This cycle consumes a significant number of platelets, which may surpass the regenerative capacity of platelets and result in thrombocytopenia.

Subgroup analysis revealed that the pressure difference between the two sides of the occluder was significantly higher in the large PDA group when compared to the small-medium PDA group. Additionally, the incidence of thrombocytopenia in the large PDA group was 64.29%. The larger occluder diameter required for large PDAs leads to a substantial increase in pressure difference on both sides of the occluder, thereby increasing the risk of severe thrombocytopenia after the operation. This mechanism can be attributed to two factors. Firstly, in clinical practice, PDAs are often anatomically funnel-shaped and tubular, resulting in a longer and columnar waist of the PDA occluder. The polyester fiber in the PDA occluder has a strong adhesive property for platelets [21], and large occluder has a greater contact area with circulating blood flow for platelet adhesion. Further, platelet aggregation reaction consists of two phases: a rapid and reversible phase I, and a slow and irreversible phase II [22]. The polyester fiber in the PDA occluder has sparse flow resistance, and larger occluders have more small cavities in the polyester fiber layer. After the release of the occluder umbrella, phase I platelet aggregation may occur. However, due to the high pressure difference on both sides of the occluder, phase I aggregation is susceptible to disruption by high-speed blood flow. The scattered platelets are mechanically damaged by the metal mesh and small cavities due to the high-speed blood flow, leading to significant platelet consumption.

Several studies have demonstrated a correlation between thrombocytopenia and residual shunt after interventional PDA occlusion [14, 23]. The high-speed blood flow passing through the residual shunt, whether located at the edge or center of the occluder umbrella, is likely to cause mechanical damage to blood vessels and platelet thrombus formation [24]. Our logistic regression analysis also confirmed that residual shunt is a risk factor for a reduction in platelet count after PDA occlusion. Although only 6 patients had a residual shunt after PDA occlusion, all of them experienced severe platelet count reduction. Platelet count in the patients dropped to the lowest level on days 4–6 after transcatheter PDA occlusion. Thus, we reason that residual shunt plays a significant role in the development of severe platelet count reduction following PDA closure.

The PDA occluder commonly used in clinical practice is composed of a highly elastic memory nickel titanium alloy in a mushroom-shaped reticular structure. It consists of three layers of polymer polyester fiber sheets [25]. The expanded surface area allows for increased contact with platelets, leading to platelet activation and aggregation [26, 27]. However, there is a possibility of thrombocytopenia occurring after PDA occlusion due to allergic reactions towards the polyester fibers [28]. Additionally, the installation of a PDA occluder can result in an increased concentration of nickel in the serum, and induce cause severe platelet deformation and abnormal aggregation [29, 30].

Considering the application of heparin during the occlusion operation, it cannot be ruled out that heparin may also cause thrombocytopenia. However, the direct causal relationship between heparin and thrombocytopenia is still a matter of debate. One report suggests that thrombocytopenia may be caused by heparin, possibly due to its inappropriate use during surgery [31]. Most studies indicate that the amount of heparin used during the operation is only a contributing factor to the decrease in platelet count [23, 32]. Another report claims that the amount of heparin used in transcatheter occlusion is not related to the decrease in platelet count [28]. The diagnosis of heparin-induced thrombocytopenia primarily relies on the detection of 4Ts and heparin/PF4 antibody, although its specificity and sensitivity are not high [33]. In our study, all patients were administered with 100U/kg heparin during the operation. Our analysis suggests that there was no significant difference in heparin dosage between the normal platelet count group and the thrombocytopenia group. Logistic regression analysis indicated no correlation between the dosage of heparin and the decrease in platelet count. Therefore, we did not consider heparin as a primary cause for thrombocytopenia after PDA occlusion operation.

## Conclusions

Thrombocytopenia is a common complication observed in PDA patients after transcatheter occlusion surgery. Our retrospective analysis revealed that the diameter of the occluder, the pressure difference between the two sides of the occluder, and the presence of residual shunt are significant risk factors associated with the occurrence of postoperative thrombocytopenia. However, it is crucial to conduct a multicenter and long-term prospective study to further assess the prognosis of PDA patients with thrombocytopenia after transcatheter occlusion. Additionally, percutaneous closure of large PDA have a higher risk of thrombocytopenia, which requires close and long-term follow-up to avoid adverse events.

## Data Availability

The data used to support the findings of this study are included within the article.
